# Supplementary material for: Optimization of differentiation and transcriptomic profile of THP-1 cells into macrophage by PMA
Source: PLoS One. 2023 Jul 17;18(7):e0286056. doi: 10.1371/journal.pone.0286056 (PMC10351730; doi:10.1371/journal.pone.0286056)
Supplement: S1 File — (ZIP) [file pone.0286056.s001.zip › supporting files/RNA-seq report.zip!/RNA-seq report/1_QC_result/GC/control-2_base_content_contribution.pdf]

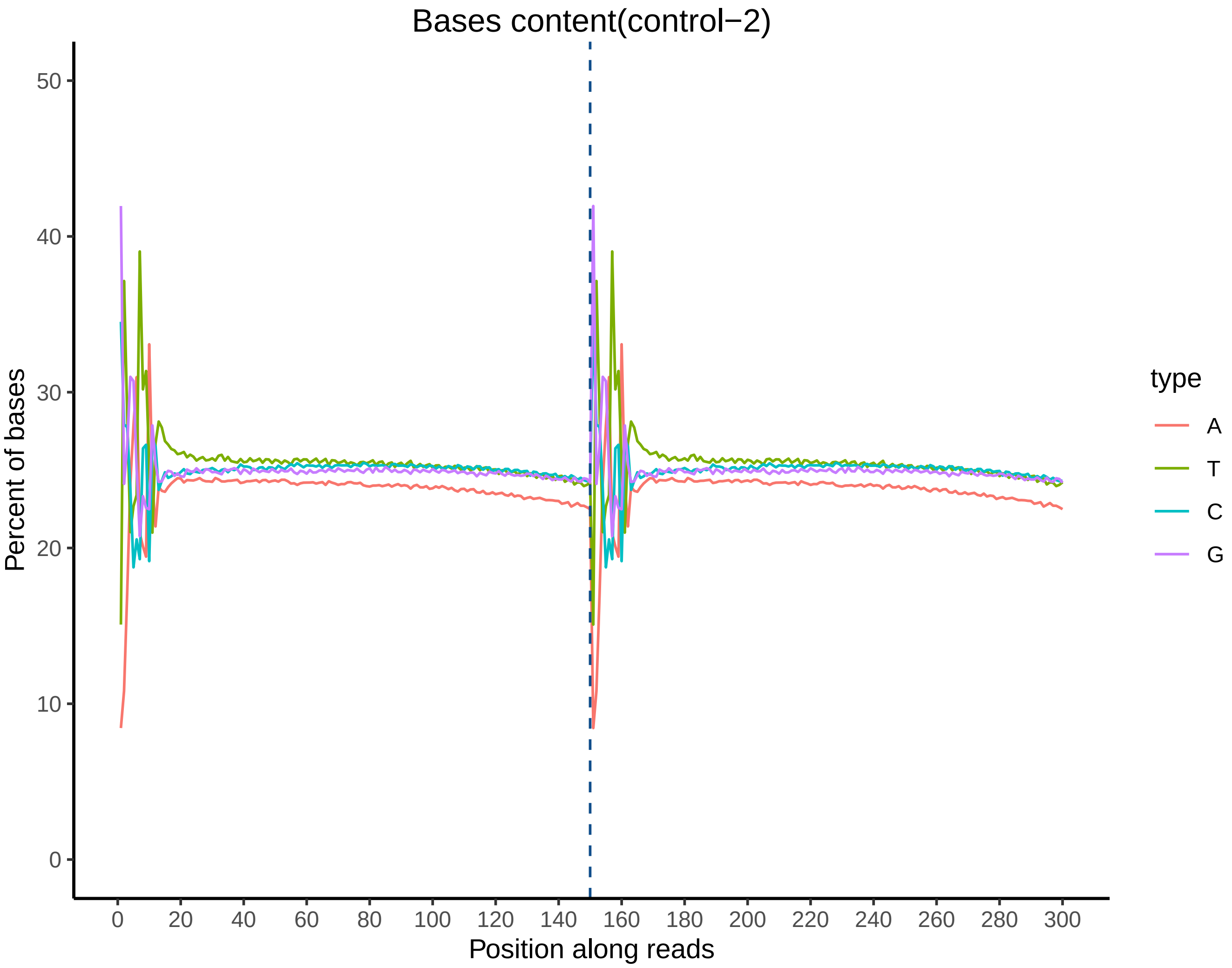

Bases content(control−2)
Percent of bases
0
10
20
30
40
50
0
20
40
60
80
100
120
140
160
180
200
220
240
260
280
300
Position along reads
type
A
T
C
G
